# The mediating effect of achievement motivation on mindfulness and procrastination behavior of nursing students: A correlational study

**DOI:** 10.1097/MD.0000000000033327

**Published:** 2023-03-24

**Authors:** Meng Lina, Li Yang, Guan Qing

**Affiliations:** a Department of Nursing, Harbin Medical University, Daqing, China; b Department of Education, Harbin Medical University, Daqing, China.

**Keywords:** achievement, mindfulness, procrastination behavior

## Abstract

Procrastination behavior is prevalent among nursing students in China. However, little research has examined how mindfulness is associated with procrastination behavior, via achievement motivation among nursing students. The aims of this study were to investigate the relationship between procrastination behavior, mindfulness and achievement motivation, and explore the mediating effect of achievement motivation on mindfulness and procrastination behavior of nursing students. A correlational cross-sectional study was performed using an online questionnaire. The study was conducted from January to March 2022 among 632 students from 1 university. A general information questionnaire, Achievement Motivation Scale, Five Facet Mindfulness Questionnaire and General Procrastination Scale (GPS) were used for investigation. Calculations were performed using SPSS Statistics, version 25. Descriptive statistics, correlation, and process plug-in mediation effect analyses were used to analyze the data. A total of 640 questionnaires were issued and 632 valid questionnaires were finally recovered, with an effective recovery rate of 98.75%. The GPS score of 632 nursing undergraduates was (55.80 ± 6.57), achievement motivation scale score was (−2.49 ± 8.73), Five Facet Mindfulness Questionnaire score was (118.21 ± 18.39). Achievement motivation and psychological capital were all negatively correlated with procrastination behavior (*r* = −0.291, −0.483; *P* < .01). Achievement motivation played a partial mediating role between mindfulness and procrastination behavior, and the mediating effect accounted for 59.82% of the total effect. The procrastination behavior of nursing undergraduates is at the middle level. Mindfulness can influence procrastination behavior through achievement motivation. Measures are needed to decrease the procrastination behavior by developing mindfulness programs to increase their achievement motivation.

## 1. Introduction

Procrastination behavior refers to the phenomenon that individuals, when faced with a task that must be completed, cannot immediately engage in it and finish it on time, but intentionally engage in other behaviors unrelated to it and postpone the task.^[1]^ Procrastination will not only hinder the achievement of learning and work goals, but also long-term procrastination will lead to anxiety, depression, learned helplessness and other negative emotions, and the accumulation of negative emotions will lead to a series of physical or psychological problems.^[2]^ It has been reported that 96.1% of medical students have different degrees of procrastination, and 45.3% of them are moderate to severe, which is a group with serious procrastination among college students.^[3]^ As a member of medical students, nursing students also have academic procrastination, with an incidence of 46% to 87%.^[4]^ Long time procrastination behavior will not only lead to lower academic performance and hinder self-development of nursing students, but also cause serious damage to their quality of life and mental health.^[5]^

Previous studies have indicated that the occurrence of procrastination was the result of a variety of factors, such as personality traits, external environment and task characteristics.^[6]^ Achievement motivation is the internal motivation of individuals to set high standards for themselves, motivate themselves to pursue excellence and achieve the set goals, and is an important predictor of reducing the occurrence of procrastination in college students.^[7]^ Evidence showed that the desire for success was negatively correlated with procrastination, while procrastination was positively correlated with failure avoidance.^[8]^ Li et al found that students with higher achievement motivation procrastinated less.^[9]^ Individuals with high achievement motivation tend to set difficult goals for themselves, and often get achievements and satisfaction from completing challenging tasks. Driven by such intrinsic motivation, they often experience pleasure in the process of learning, thus reducing the possibility of procrastination.^[9]^ However, how achievement motivation is linked with procrastination is still poorly understood.

Previous studies show that achievement motivation is closely related to psychological factors, and mindfulness is one of them.^[10]^ Mindfulness is the nonjudgmental observation of internal and external stimuli, that is, the current attention is focused on the individual internal or external environment experience.^[11,12]^ Due to individual differences in the tendency to maintain mindfulness in daily life, mindfulness has also been regarded as a personality trait.^[13]^ Studies have found that mindfulness has significant positive effects on individuals’ physical health, cognitive, emotional and behavioral problems, and interpersonal adaptation.^[14,15]^ Recent studies have confirmed that a mindfulness state can positively predict the achievement motivation at the workplace.^[16,17]^

Focus on procrastination behavior among college students has been increasing in recent years.^[18]^ Some studies believe that when individuals have a high achievement motivation, their procrastination will be reduced, but some students have a strong motivation to pursue success, but lack good time management and self-control, so they procrastinate more.^[19]^ At present, few studies have explored the relationship between mindfulness, achievement motivation and procrastination.

**Figure 1.**
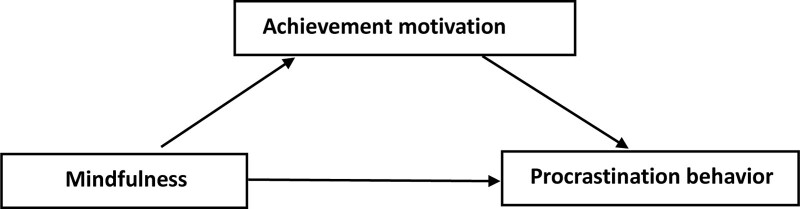
Conceptual model of mindfulness, achievement motivation and procrastination behavior.

To our best knowledge, this study appears to be the first to analyze the associations between mindfulness, achievement motivation and procrastination behavior in undergraduate nursing students. For this reason, for the lack of studies reporting on procrastination behavior in undergraduate nursing students, and due to the importance of procrastination in their study and future jobs, it seems important to study the factors which may influence it in the ambit of procrastination behavior. Based on the existing literature, a conceptual model involving mindfulness, achievement motivation and procrastination behavior is proposed, as shown in Figure 1, to examine the meditation pathway between mindfulness and procrastination behavior. Specifically, we hypothesize that: Mindfulness is negatively associated with procrastination behavior in undergraduate nursing students in China; Achievement motivation is negatively associated with procrastination behavior in undergraduate nursing students; Mindfulness is negatively associated with achievement motivation; The effect of mindfulness on procrastination behavior is mediated by achievement motivation in nursing students.

## 2. Methods

### 2.1. Design and sample

This descriptive correlation study was designed to examine the relationship among mindfulness, achievement motivation and procrastination behavior in undergraduate nursing students. A convenience sample was used to select nursing students from January to March 2022. The inclusion criteria were students majoring in nursing, willing to participate, and being able to complete the questionnaires. Exclusion criteria included: dropped out of school or a temporary absence from school. The sample size was calculated according to the literature that the medium required sample size to detect the mediated effect was 200 participants.^[20]^

### 2.2. Instruments

The basic demographic questionnaire: This study collected 7 demographic characteristics which included age, gender, grade, residence, whether from single-child family, whether they served as class leader, and whether they were interested in their major.

General Procrastination Scale (GPS): The GPS was compiled by Lay in 1986 and revised by Chu into Chinese version in 2010.^[21]^ The scale includes 20 items, and each item is scored on a 5-point Likert scale ranging from 1 (completely inconsistent) to 5 (completely consistent). The total score ranges from 20 to 100, and the higher the score is, the more serious the procrastination behavior. The scale has good reliability and validity.^[21]^ The Cronbach α was 0.86 in the previous study^[21]^ and 0.88 in this study.

Achievement Motivation Scale: The achievement motivation scale was compiled by Gjesme and Nygard in 1970 and revised by Ye and Hagtvet into Chinese version in 1992.^[22]^ The scale includes 30 items, which are divided into 2 dimensions: pursuing success and avoiding failure. Each item is scored on a 4-point Likert scale ranging from 1 (completely inconsistent) to 4 (completely consistent). The total score is the difference between the score of pursuing success and the score of avoiding failure which ranges from −45 to 45, and the higher the score is, the stronger achievement motivation is. The scale has good reliability and validity.^[22]^ The Cronbach α was 0.91 in the previous study^[22]^ and 0.90 in this study.

Five Facet Mindfulness Questionnaire: The Five Facet Mindfulness Questionnaire was compiled by Baer in 2006 and revised by Deng into Chinese version in 2011.^[23]^ The scale includes 39 items, and each item is scored on a 5-point Likert scale ranging from 1 (completely inconsistent) to 5 (completely consistent). The total score ranges from 39 to 195, and the higher the score is, the more mindfulness state. The scale has good reliability and validity.^[23]^ The Cronbach α was 0.84 in the previous study^[23]^ and 0.86 in this study.

### 2.3. Data collection

After obtaining the consent of the relevant department, an online meeting was held to explain the purpose and method of filling the questionnaire in the unified instruction, and the questionnaire was anonymous and voluntary. After online informed consent was obtained by each participant, the online survey link was sent by the researcher to all nursing students who were willing to participate in the study. All data were collected by the WenJuanXing program (a software for data collection in China). In order to improve the accuracy of questionnaire response, the electronic questionnaire has been equipped with logic check, error reminder, automatic jump and other verification procedures.

### 2.4. Data analysis

The Statistical Packages for Social Sciences, version 25 (IBM Corp., Chicago, IL) was used to analyze the data. The mean and standard deviation of procrastination behavior of different ages and grades were analyzed by descriptive statistics, independent sample T test and 1-way ANOVA. Pearson correlation test was used to investigate the relationship among mindfulness, achievement motivation and procrastination behavior. The mediation effect test model of Bootstrap was used to test and analyze the mediating effect of achievement motivation whether played a mediating role between mindfulness and procrastination behavior. A *P* value ≤ .05 was considered statistically significant.

### 2.5. Ethical considerations

This study was approved by the Ethics committee of Harbin Medical University. The researcher illustrated the aim of the study and promised that all data would be anonymized. Students could withdraw from the study at any time with any reason. An online informed consent form was completed by each participant.

## 3. Results

Initially, there were 640 students participating in this study and surveys with invalid data and missing data were eliminated. Finally, a total of 632 valid online questionnaires were included in the analysis, with an effective recovery rate of 98.75%.

### 3.1. The distribution of demographic characteristics

The average age of the participants was 20.38 ± 1.58 years with a range of 17 to 25 years. Most participants were female (84.7%). The residence in urban accounted for 61%. There were 284 participants (45.6%) from a single-child family. More than half of the participants did not have an administrative title (66.1%) and few of the participants (9.9%) were not interested in their major.

### 3.2. Comparison of GPS scores of undergraduate nursing students with different demographic characteristics

As shown in Table 1, the total score of GPS has significant differences among grades (*F* = 9.62, *P* < .01) and major interests (*F* = 19.76, *P* < .01); however, there was no significance among different gender, residence, only-child family and experience in being a student leader (*P* > .05).

**Table 1:** The difference between of different groups of GPS (N = 632).

Variables		N (%)	GPS score	*t*/*F*	*P*
Gender	Male	84 (15.3%)	55.88 ± 6.48	0.29*	.78
	Female	548 (84.7%)	56.18 ± 8.08		
Grade	Freshmen	190 (30.1%)	54.15 ± 6.33	9.62†	<.01
	Sophomore	139 (22.0%)	57.70 ± 5.40		
	Junior	190 (30.1%)	56.63 ± 6.46		
	Senior	113 (17.9%)	56.19 ± 6.52		
Residence	Urban	389 (61.0%)	55.30 ± 6.77	1.49*	.14
	Rural	243 (39.0%)	55.30 ± 6.77		
Single-child family	Yes	284 (45.6%)	55.44 ± 6.84	1.20*	.23
	No	348 (54.4%)	56.13 ± 6.20		
Served as class leader	Yes	211 (33.9%)	55.00 ± 6.49	1.14*	.26
	No	412 (66.1%)	55.74 ± 7.07		
Interested in major	Yes	178 (28.1%)	53.55 ± 7.17	19.76†	<.01
	General	392 (62.0%)	56.31 ± 6.07		
	No	62 (9.9%)	59.08 ± 5.74		

* *t* test.

† One-way analysis of variance.

### 3.3. The relationship between achievement motivation, mindfulness, and procrastination behavior of undergraduate nursing students

The mean scores of achievement motivation, mindfulness, and procrastination behavioral were found to be −2.49 ± 8.73, 118.21 ± 18.39, and 55.80 ± 6.57, respectively. Furthermore, Table 2 showed the significant negative correlations between participants’ procrastination behavioral and achievement motivation (*r* = −0.483, *P* < .01) and mindfulness (*r* = −0.291, *P* < .01). Mindfulness had a significant positive correlation with achievement motivation (*R* = 0.399, *P* < .01).

**Table 2:** Means and correlation coefficients of the variables (N = 632).

Variables	Mean ± SD	Achievement motivation (*r*)	Mindfulness (*r*)
Achievement motivation	−2.49 ± 8.73		
Mindfulness	118.21 ± 18.39	0.399*	
Procrastination behavioral	55.80 ± 6.57	−0.291*	−0.483*

SD = standard deviation.

* *P* < .01

### 3.4. The mediating role of achievement motivation in the relationship between mindfulness and procrastination

In the regression analysis, we set procrastination behavioral as the dependent variable and mindfulness as the independent variable and achievement motivation as mediating variable. The results showed that the Bootstrap 95% confidence intervals of the direct and indirect effects of achievement motivation on procrastination did not contain 0, indicating that achievement motivation played a partial mediating role between mindfulness and procrastination, with an explanatory power of 59.82%. The results of Standardized Total Effect (*β* = −0.219) and Standardized Direct Effect (*β* = −0.088) are shown in Table 3.

**Table 3:** Mediating analyze of mindfulness in the relationship between achievement motivation and procrastination (N = 632).

	*β*	SE	95% CI		Account
			Lower	Upper	
Total effect	−0.219	0.029	−0.275	−0.162	
Direct effect	−0.088	0.028	−0.144	−0.032	40.18%
Indirect effect	−0.131	0.019	−0.169	−0.094	59.82%

## 4. Discussion

To our best knowledge, this is the first study to investigate the relationship of mindfulness, achievement motivation and procrastination behavior on nursing student in China, which yields that the achievement motivation has a mediating effect between mindfulness and procrastination behavior.

### 4.1. The level of procrastination among nursing students

The score of procrastination of nursing students was 55.80 ± 6.57, which indicated a medium level. The results showed that sophomore nursing students have the highest score of procrastination, which was consistent with the research results of Zhang et al.^[24]^ The reason may be that in the second grade, nursing students are formally exposed to professional courses and nursing skills, and the rigid training of schoolwork pressure and operation causes their enthusiasm to fade, resulting in fatigue and boredom, and professional maladjustment, which affects the internal learning motivation and leads to the occurrence of procrastination.^[25]^ The score of procrastination of nursing students not interested in the nursing major were higher than that of nursing students who chose nursing majors voluntarily. Students who voluntarily choose nursing major have a strong interest in nursing and correct cognition, high learning enthusiasm, and can timely and actively complete the planned tasks.^[26]^ However, students who are not interested in the nursing major have a low enthusiasm for nursing, poor stability of professional thinking and learning enthusiasm, and are more prone to procrastination.^[27]^ Therefore, nursing educators should pay attention to the teaching of clinical skills, organize flexible nursing professional skills courses, stimulate students’ interest in learning, improve students’ clinical work competitiveness, and reflect their own professional value.

### 4.2. Achievement motivation has a negative predictive effect on procrastination

The results showed that achievement motivation has a negative predictive effect on procrastination, which was consistent with the research results of Jia et al.^[28]^ Individuals with high achievement motivation will actively engage themselves in the work tasks that they feel are valuable and can give them a sense of achievement and satisfaction, thus reducing the probability of procrastination.^[29]^ A high level of achievement motivation can enable nursing students to acquire more positive emotions and maintain a high expectation level for completing tasks, so as to prompt them to complete tasks in time and reduce the occurrence of procrastination.^[30]^ It is suggested that nursing educators can set up reward programs to stimulate students’ learning enthusiasm and reduce procrastination behavior by enhancing achievement motivation. In addition, teachers should guide students to make plans according to the time and the difficulty of tasks to improve their time management ability, and gradually enhance the sense of achievement through the progress of tasks completed.

### 4.3. Mindfulness has a negative predictive effect on procrastination

Mindfulness has a negative predictive effect on procrastination, which was consistent with previous research.^[31]^ The higher the level of mindfulness, the lower level of procrastination behavior. Mindfulness has a significant positive effect on individual mental health which can help nursing students enhance their adaptability and initiative, thus reducing procrastination.^[10]^ With increasing the level of mindfulness, the individual self-esteem will also improve, thus helping individuals in academic, work and life aspects produce high self-efficacy, allowing the individual to effortlessly deal with a series of problems, and thus improves the procrastination behavior.^[32]^ Nursing students with high level of mindfulness tend to be flexible in regulating and maintaining attention to current experiences and are more likely to engage in current activities. They can focus on sticking to the goal and improving performance, and thus reduce their procrastination behavior.^[33]^ It is suggested that nursing educators should pay attention to students’ mental health and provide adequate and effective mental health resources to help nursing students establish a mindfulness state, thus having a more positive life attitude and sense of goal and fulfillment to alleviate the procrastination behavior.

### 4.4. Achievement motivation plays a mediating role between Mindfulness and procrastination behavior

This study found that achievement motivation played a partial mediating role between mindfulness and procrastination behavior, that is, mindfulness can directly affect procrastination behavior of nursing students, and also can indirectly affect procrastination behavior through achievement motivation. The nonjudgmental part of mindfulness is less likely to make individuals resent the task, and its awareness and attention components are less likely to lead to loss of self-control and distraction, which play a direct role in improving procrastination.^[34]^ In addition, we believe that the open, accepting and nonjudgmental attitude contained in mindfulness can enable individuals to truthfully accept themselves and their relationships with the outside world, which may make individuals less susceptible to negative cognition and emotions in the face of pressure and difficulties, thus helping individuals to form a more objective and positive self-evaluation (core self-evaluation), and thus more confident in their own abilities. Improving achievement motivation, thereby improving procrastination, is the way to go.^[35]^ The above results not only support the effect of mindfulness training on improving procrastination of college students, but also show the mechanism and approach of mindfulness training.^[34]^ As a positive psychological advantage, mindfulness can help nursing students enhance their achievement motivation, thus reducing procrastination.^[12]^ Nurse educators should strengthen the development and cultivation of mindfulness and achievement motivation, carry out intervention training in the way of team communication and cooperation, so as to strengthen the mindfulness and achievement motivation of nursing students and reduce procrastination behavior.

### 4.5. Limitations

Although this study identified the relationship of nursing students’ mindfulness, achievement motivation and procrastination behavior, there were still limitations. Firstly, the participants of this study were recruited from 1 university so the results cannot be generalized due to the sample selection method and data collection method, and readers need to raise caution when applying our findings to nursing students from other countries or from different cultural backgrounds. Further studies should be conducted on larger samples in different cultures. Moreover, the self-report nature of the measures mean that responses are subject to bias and socially desirable responding which may not avoid the deliberate withholding or perfunctory responses by respondents.

## 5. Conclusion

The results in this study suggested that the procrastination behavior of nursing students was high and was negatively associated with the achievement motivation and mindfulness. Moreover, this study confirmed the mediating effect of achievement motivation on mindfulness and procrastination behavior in nursing students. The results of this study could help nurse educators better understand the relationship between achievement motivation and mindfulness and procrastination behavior. It is suggested that nursing educators ought to assist students in cultivating mindfulness as a means to improve the achievement motivation and thus decrease the procrastination behavior.

## Acknowledgments

All authors were grateful for all the participants and all student counselors in this study for their cooperation.

## Author contributions

**Conceptualization:** Meng Lina, Li Yang, Guan Qing.

**Formal analysis:** Meng Lina, Li Yang, Guan Qing.

**Funding acquisition:** Meng Lina, Li Yang, Guan Qing.

**Investigation:** Li Yang.

**Methodology:** Meng Lina, Li Yang, Guan Qing.

**Project administration:** Meng Lina, Li Yang.

**Resources:** Guan Qing.

**Supervision:** Li Yang, Guan Qing.

**Validation:** Meng Lina.

**Writing – original draft:** Meng Lina, Li Yang, Guan Qing.

**Writing – review & editing:** Meng Lina, Li Yang, Guan Qing.
